# Supplementary material for: Genome-wide divergence among invasive populations of Aedes aegypti in California
Source: BMC Genomics. 2019 Mar 12;20:204. doi: 10.1186/s12864-019-5586-4 (PMC6417271; doi:10.1186/s12864-019-5586-4)
Supplement: Supplementary file 3 — Figure S1. Genome-wide comparison of Ae. aegypti populations comparing GC2 vs GC3, GC2 vs GC4, GC3 vs GC4, and GC1 vs GC1-B (Vero Beach and Exeter). (PDF 352 kb) [file 12864_2019_5586_MOESM3_ESM.pdf]

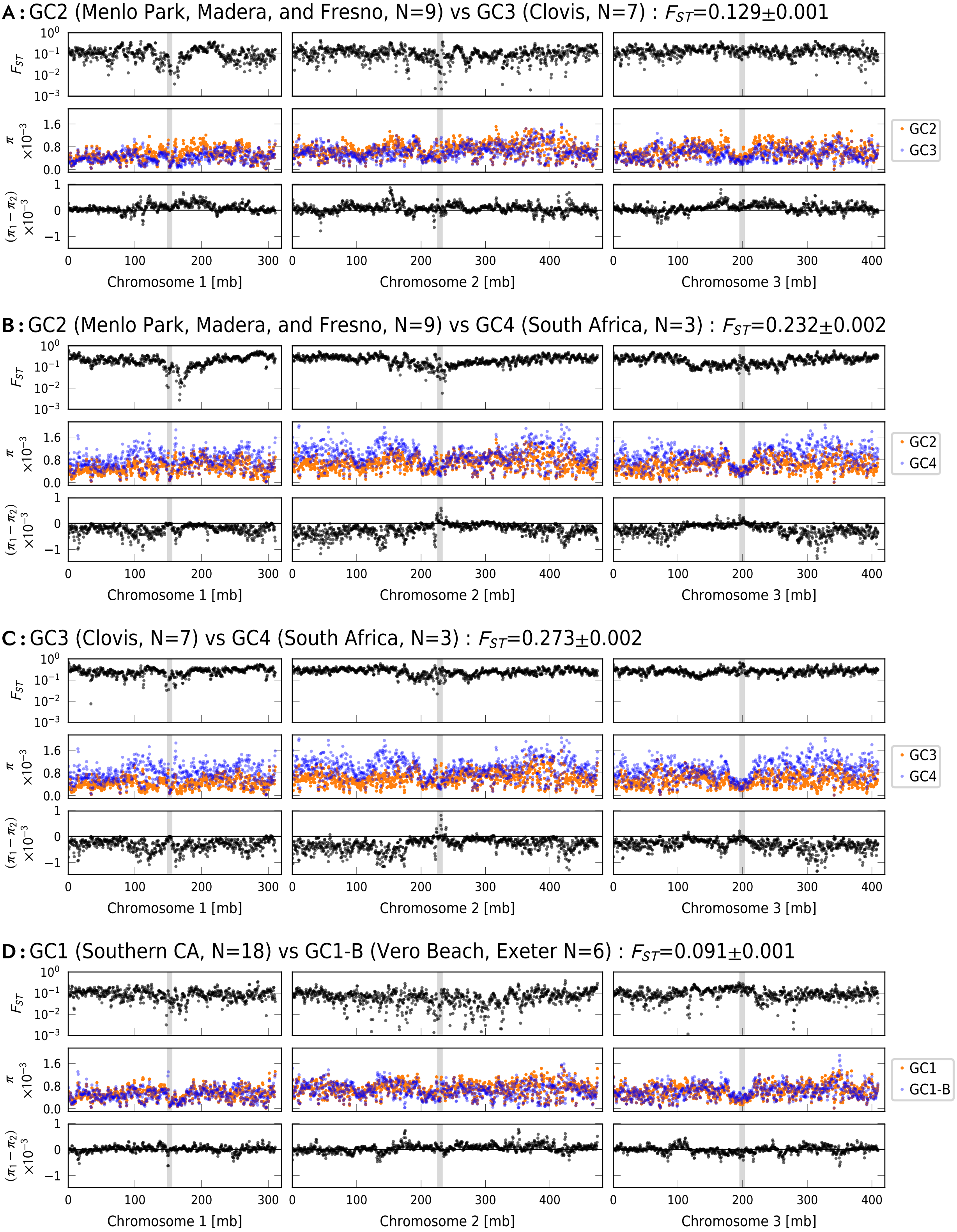
A : GC2 (Menlo Park, Madera, and Fresno, N=9) vs GC3 (Clovis, N=7) : F_{ST}=0.129±0.001
B : GC2 (Menlo Park, Madera, and Fresno, N=9) vs GC4 (South Africa, N=3) : F_{ST}=0.232±0.002
C : GC3 (Clovis, N=7) vs GC4 (South Africa, N=3) : F_{ST}=0.273±0.002
D : GC1 (Southern CA, N=18) vs GC1-B (Vero Beach, Exeter N=6) : F_{ST}=0.091±0.001
F_{ST}
10^0
10^{-1}
10^{-2}
10^{-3}
π $\times 10^{-3}$
1.6
0.8
0.0
$(\pi_1 - \pi_2)$ $\times 10^{-3}$
1
0
−1
GC2
GC3
GC4
GC1
GC1-B
Chromosome 1 [mb]
Chromosome 2 [mb]
Chromosome 3 [mb]
0
100
200
300
400
