# Supplementary material for: Genome-wide divergence among invasive populations of Aedes aegypti in California
Source: BMC Genomics. 2019 Mar 12;20:204. doi: 10.1186/s12864-019-5586-4 (PMC6417271; doi:10.1186/s12864-019-5586-4)
Supplement: Supplementary file 4 — Appendix S1. Comparison of Evans et al. 2015 SNPchip probe sequences and genotypes with our data. (DOCX 554 kb) [file 12864_2019_5586_MOESM4_ESM.docx]

**Additional file 4: Appendix S1 – Comparison of Evans et al. 2015 SNPchip probe sequences and genotypes with our Illumina Next-generation sequencing data.**

We saw a golden opportunity to narrow down the source populations of California *Aedes aegypti* by integrating the SNPchip genotype data from Evans et al. 2015 which includes *Aedes aegypti* individuals worldwide including two countries in Africa, Australia, Thailand, Brazil, French Polynesia, Mexico. In order to call genotypes of 25,589 biallelic SNPs used in Evans et al. (2015) study, we blasted the SNP probe sequences provided as supplementary data of Evans et al. (2015) paper. Of note, the SNPchip contains total of 50,000 SNPs but Evans et al. filtered out for various criteria and used the final set of 25,589 SNPs for their analysis.

The 71bp-long SNP probe sequences containing a target SNP and its flanking sequences were based on AaegL1 reference. Thus, we blasted the probe sequence to the AaegL5 reference we used for analysis to find the geographic coordinates and the strand orientation. We filtered the BLAST results in the most stringent way by excluding (1) all hits with more than 1 mismatch, (2) all hits that were shorter than the query (71 bp), (3) all hits with gap openings, and (4) all hits on unplaced scaffolds (NIGP). From the full list of 25,298 probe sequences 4,981 (19.7%) dropped out, meaning there is not a single hit in the genome fulfilling these criteria. From the remaining 20,317 probe sequences only 191 (0.9%) had multiple hits with identical quality. Highest number of hits for a probe sequence was 8. This means 79.6% of all probes have perfect BLAST hits without equally good secondary ones. If we used the relaxed criteria of 95% sequence similarity, there are 3,015 (11.9%) sequences with multiple matches. Highest number of hits for a probe sequence was 98.

The following series of images shows examples of (1) genotype discrepancies, (2) sequence differences (arising from indel mutations), (3) non-biallelic SNPs, (4) polymorphisms surrounding the target SNPs that resulted in mismatched genotype calls between the SNPchip and Illumina next-generation sequencing platforms.

1. Genotype discrepancy example: AX-93242921

Probe sequence: TCAGTTCGGTTTTTGTTGGCAAGTGCTGCATTGTT[C/T]CGATGAGGTGAGTAGCAGCAGTACGCTTTCGCGTT

BLAST result: single match on the chromosome 3: 107766015-107766085 positive strand. No difference was detected between AaegL1 and AaegL5 except the lack of polymorphism within our dataset (Figure S2).

|  |  |  | **Genotypes** | | |
| --- | --- | --- | --- | --- | --- |
| **Platform** | **Location** | **Sample size** | **CC** | **CT** | **TT** |
| Illumina NGS | Central CA | 22 | 100% |  |  |
| Illumina NGS | Southern CA | 18 | 100% |  |  |
| Illumina NGS | Florida | 4 | 100% |  |  |
| Illumina NGS | South Africa | 3 | 100% |  |  |
|  |  |  |  |  |  |
| SNP chip | Australia | 12 |  |  | 100% |
| SNP chip | French Polynesia | 12 |  |  | 100% |
| SNP chip | Senegal | 24 |  | 8.3% | 91.7% |
| SNP chip | Texas | 8 |  |  | 100% |
| SNP chip | Hawaii | 6 |  |  | 100% |
| SNP chip | Brazil | 40 |  |  | 100% |
| SNP chip | Uganda | 12 |  |  | 100% |
| SNP chip | Florida | 11 |  | 9.1% | 90.9% |
| SNP chip | Puerto Rico | 12 |  |  | 100% |
| SNP chip | Mexico | 11 |  |  | 100% |
| SNP chip | Thailand | 12 |  |  | 100% |


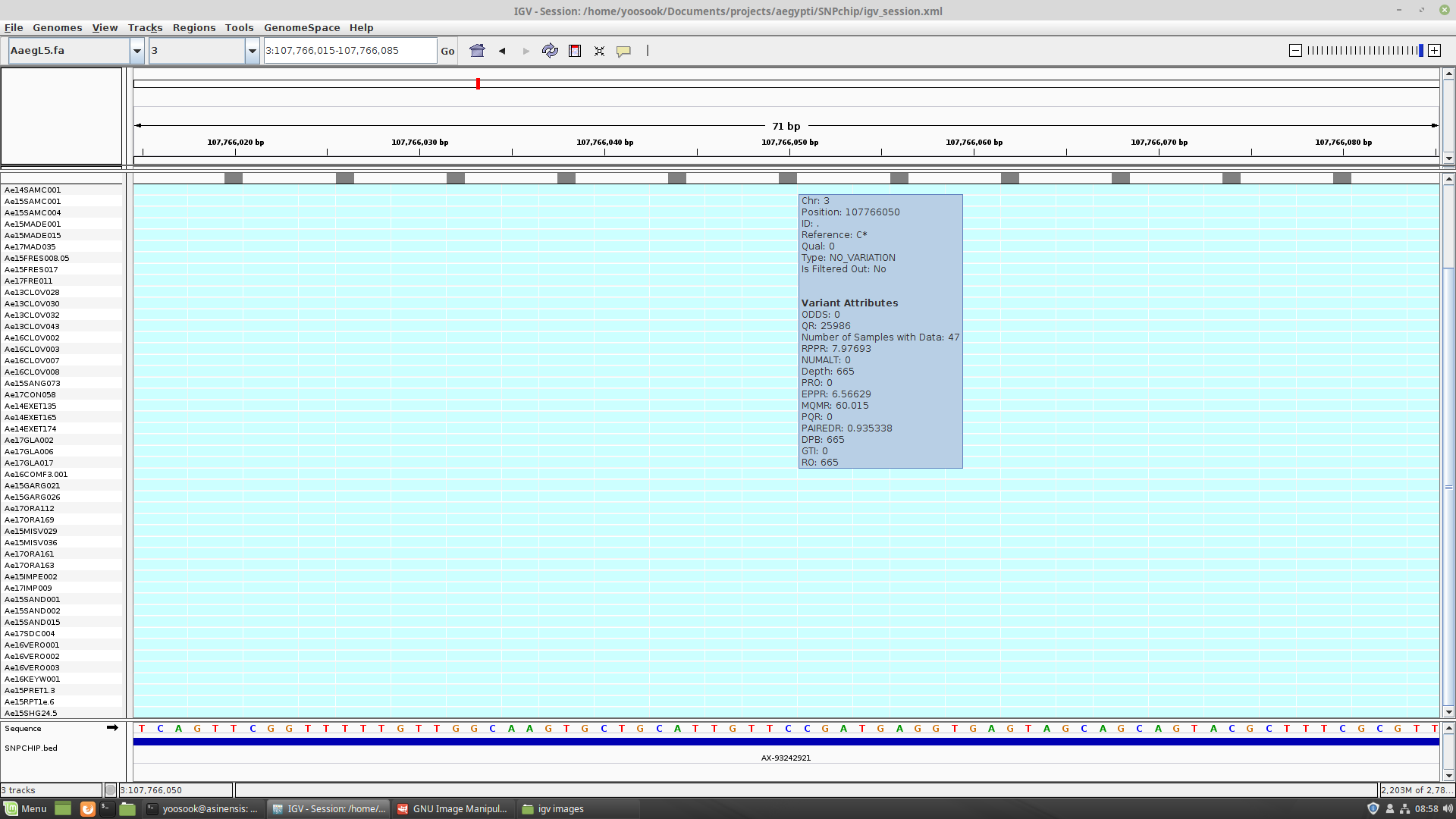


**Figure S2**: Integrated Genome Browser (IGV) view of SNP AX-93242921 showing no polymorphism surrounding target SNP. Average depth for this position was 14.1X.

1. Sequence difference + non-target polymorphism example: AX-93253298

Probe sequence: CTATCACTGACATCCTGCAGGAACGAAAGTATTCT[C/T] CATTGCTCCAGAAAAGGGGGTGATTTCGAACAGCT

AaegL5 sequence: CTATCACTGACATCCTGCAGGAACGAAAGTATTCT[C/T]TCATTGCTCCAGAAAAGGGGGTGATTTCGAACAGCT

- Probe (AaegL1) is missing T that is present in AaegL5 adjacent to the target SNP.

BLAST result: single match on the chromosome 3: 140181466-140181537 positive strand. Additional SNP near target SNP detected on some California and Florida samples (Figure S3).

| **Platform** | **Location** | **Sample size** | **CC** | **CT** | **TT** |
| --- | --- | --- | --- | --- | --- |
| Illumina NGS | Central CA | 22 |  | 13.6% | 86.4% |
| Illumina NGS | Southern CA | 18 | 5.6% | 11.1% | 83.3% |
| Illumina NGS | Florida | 4 |  | 25.0% | 75.0% |
| Illumina NGS | South Africa | 3 |  | 33.3% | 66.7% |
|  |  |  |  |  |  |
| SNP chip | Australia | 12 | 75.0% | 25.0% |  |
| SNP chip | French Polynesia | 12 | 75.0% | 25.0% |  |
| SNP chip | Senegal | 24 | 100% |  |  |
| SNP chip | Texas | 8 | 50.0% | 25.0% | 25.0% |
| SNP chip | Hawaii | 6 | 100% |  |  |
| SNP chip | Brazil | 40 | 55.0% | 30.0% | 15.0% |
| SNP chip | Uganda | 12 | 100% |  |  |
| SNP chip | Florida | 11 | 50.0% | 40.0% | 10.0% |
| SNP chip | Puerto Rico | 12 | 91.7% | 8.3% |  |
| SNP chip | Mexico | 11 | 54.5% | 45.5% |  |
| SNP chip | Thailand | 12 | 50.0% | 50.0% |  |


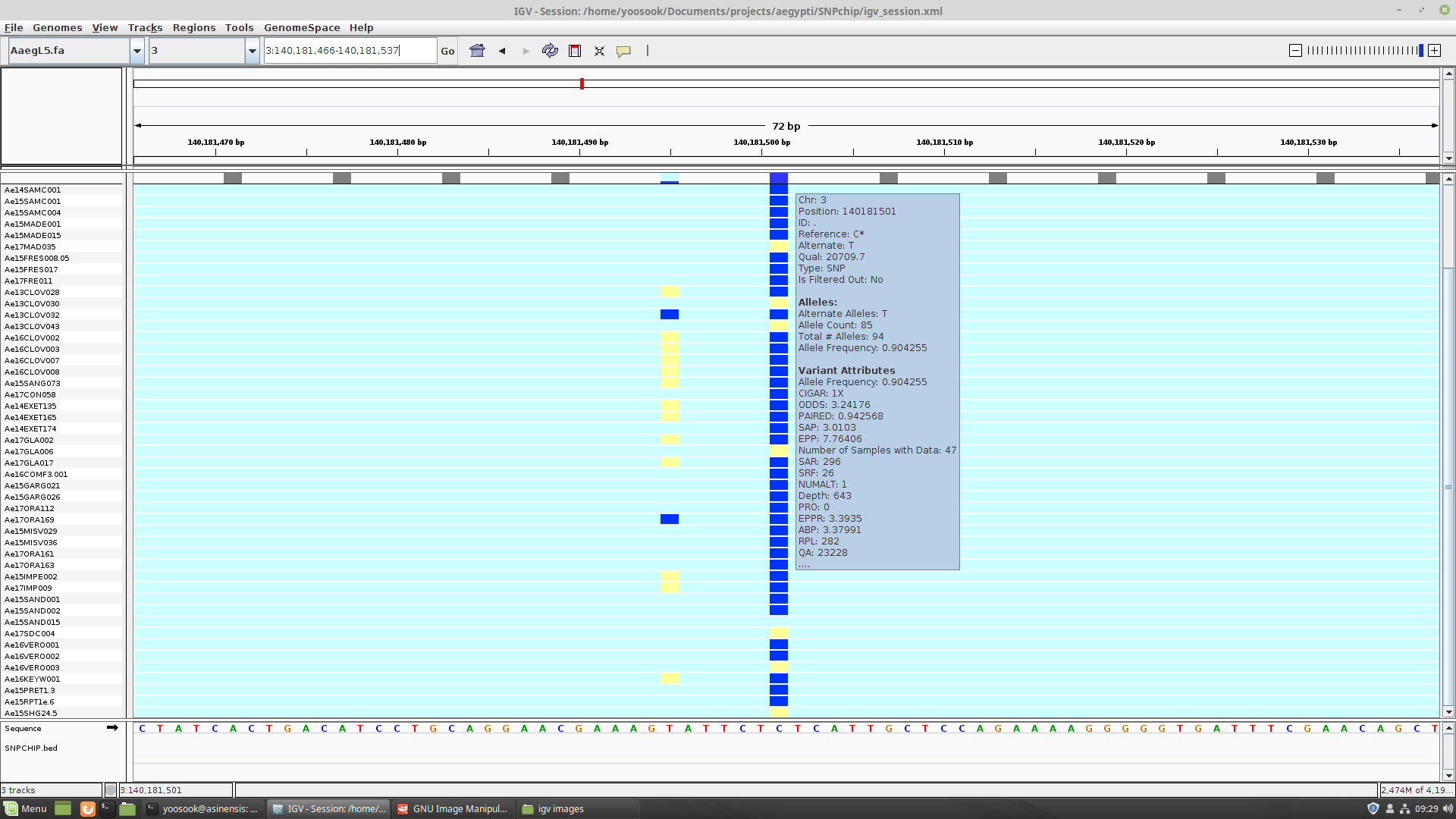


**Figure S3**: IGV view of SNP AX-93253298 showing the target SNP as well as additional non-target SNP. Average depth for this position was 13.7X.

1. Non-biallelic SNPs example : AX-93220517

Probe sequence: CGACGATCAAACATGCCAATACAACCAACACACCA[C/T] CGTTGAATTCAACATACAATACGGTTGTTGGCTTG

AaegL5 sequence: CGACGATCAAACATGCCAATACAACCAACACACCA[T/C/A]MGTTGAATTCAACATACAATACGGTTGTTGGCTTG

BLAST result: single match on the chromosome 2: 86797244-86797314 positive strand. Additional polymorphisms detected in our field samples (Figure S4).

| **Platform** | **Location** | **Sample size** | **CC** | **CT** | **TT** | **AT** | **AC** |
| --- | --- | --- | --- | --- | --- | --- | --- |
| Illumina NGS | Central CA | 22 | 100% |  |  |  |  |
| Illumina NGS | Southern CA | 18 | 100% |  |  |  |  |
| Illumina NGS | Florida | 4 | 25.0% |  |  | 25.0% | 50.0% |
| Illumina NGS | South Africa | 3 | 66.7% | 33.3% |  |  |  |
|  |  |  |  |  |  |  |  |
| SNP chip | Australia | 12 |  |  | 100% |  |  |
| SNP chip | French Polynesia | 12 |  |  | 100% |  |  |
| SNP chip | Senegal | 24 |  | 20.8% | 79.2% |  |  |
| SNP chip | Texas | 8 |  |  | 100% |  |  |
| SNP chip | Hawaii | 6 |  | 33.3% | 66.7% |  |  |
| SNP chip | Brazil | 40 |  | 2.5% | 97.5% |  |  |
| SNP chip | Uganda | 12 |  | 41.7% | 58.3% |  |  |
| SNP chip | Florida | 11 |  | 22.2% | 77.8% |  |  |
| SNP chip | Puerto Rico | 12 |  | 8.3% | 91.7% |  |  |
| SNP chip | Mexico | 11 |  |  | 100% |  |  |
| SNP chip | Thailand | 12 | 8.3% | 16.7% | 75.0% |  |  |


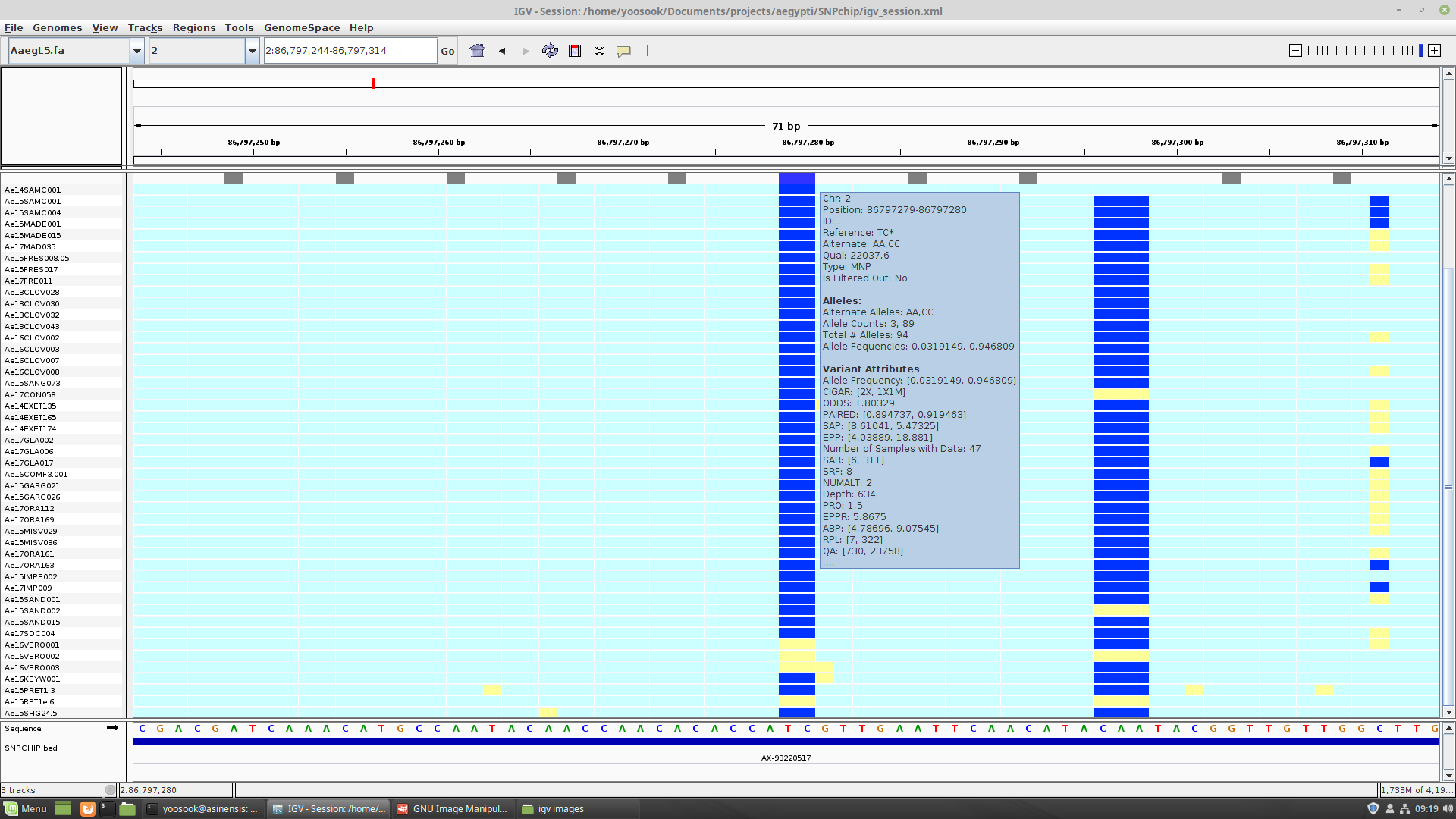


**Figure S4**: IGV view of SNP AX-93220517 showing the target SNP having three alleles – T, A and C. Additional non-target polymorphism is also observed. Average depth for this position was 13.5X.

1. Non-target polymorphism example: AX-93246466

Probe sequence: TATTTGATTTTGTATGCAAGCGATGCACTCGAGAA[A/T]GAATCAATCAAACTCTAACATTTGCACAGTCATAG

BLAST result: single match on the chromosome 1: 2743457-2743527 positive strand. Additional SNP indel near target SNP detected on some samples (Figure S5).

| **Platform** | **Location** | **Sample size** | **AA** | **AT** | **TT** | **AC** | **CT** |
| --- | --- | --- | --- | --- | --- | --- | --- |
| Illumina NGS | Central CA | 22 | 59.1% | 36.4% | 4.5% |  |  |
| Illumina NGS | Southern CA | 18 | 61.1% | 16.7% |  | 16.7% | 5.5% |
| Illumina NGS | Florida | 4 | 50.0% | 25.0% |  | 25.0% |  |
| Illumina NGS | South Africa | 3 | 100% |  |  |  |  |
|  |  |  |  |  |  |  |  |
| SNP chip | Australia | 12 |  | 8.3% | 91.7% |  |  |
| SNP chip | French Polynesia | 12 | 16.6 | 41.7% | 41.7% |  |  |
| SNP chip | Senegal | 24 | 91.6% | 4.2% | 4.2% |  |  |
| SNP chip | Texas | 8 | 50.0% | 50.0% |  |  |  |
| SNP chip | Hawaii | 6 | 16.7% | 16.7% | 66.6% |  |  |
| SNP chip | Brazil | 40 | 48.7% | 41.0% | 10.3% |  |  |
| SNP chip | Uganda | 12 | 100% |  |  |  |  |
| SNP chip | Florida | 11 | 54.5% | 36.4% | 9.1% |  |  |
| SNP chip | Puerto Rico | 12 | 25% | 66.7% | 8.3% |  |  |
| SNP chip | Mexico | 11 | 18.2% | 72.7% | 9.1% |  |  |
| SNP chip | Thailand | 12 | 83.3% | 16.7% |  |  |  |


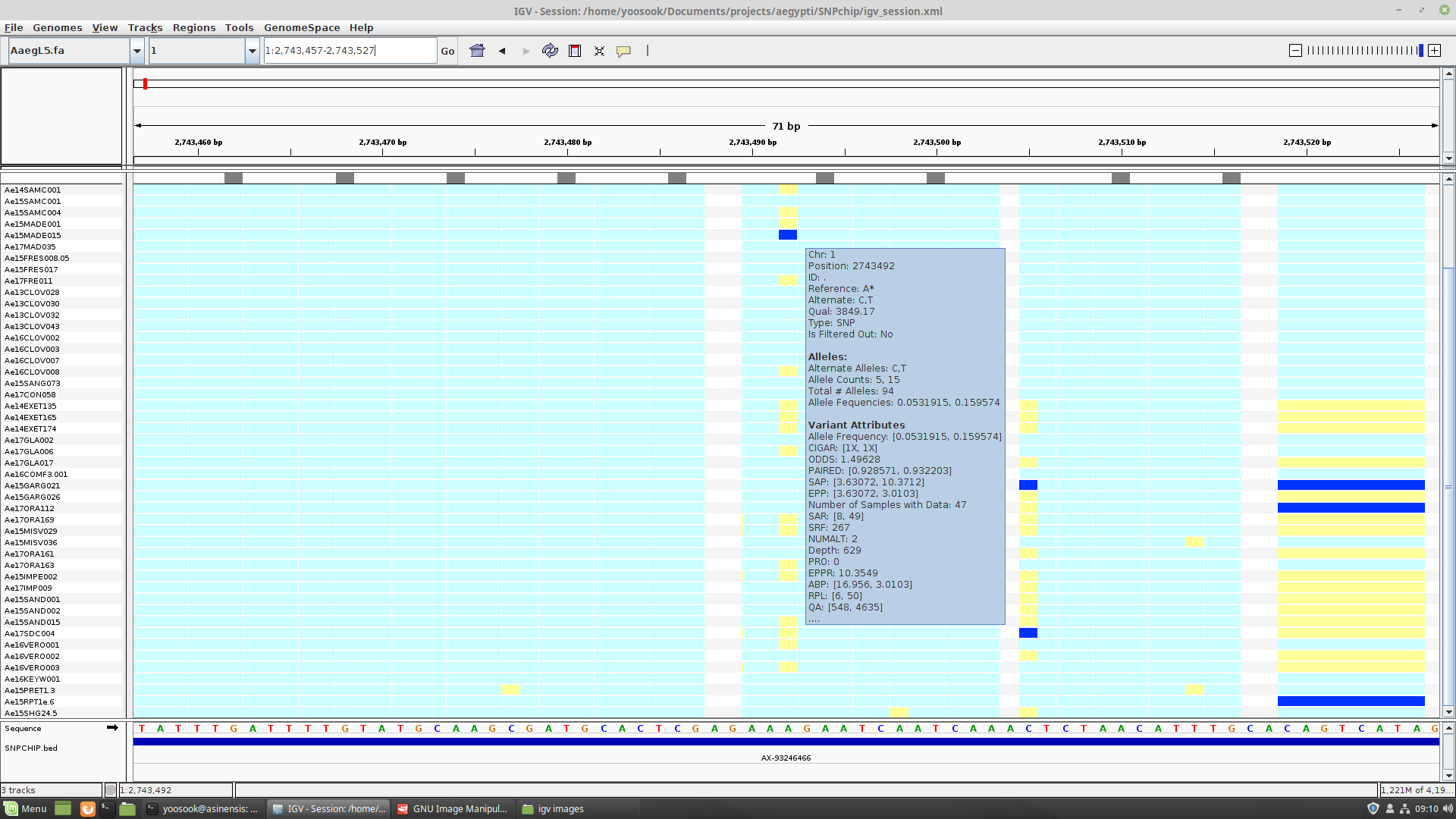


**Figure S5**: IGV view of SNP AX-93246466 showing the target SNP as well as additional non-target SNP and indels. Average depth for this position was 13.4X.
